# Longitudinal Behavior of Left-Ventricular Strain in Fetal Growth Restriction

**DOI:** 10.3390/diagnostics13071252

**Published:** 2023-03-27

**Authors:** Carla Domínguez-Gallardo, Nuria Ginjaume-García, Johana Ullmo, Antonio Fernández-Oliva, Juan Parra, Ana Vázquez, Mónica Cruz-Lemini, Elisa Llurba

**Affiliations:** 1 Department of Obstetrics and Gynaecology, Institut d’Investigació Biomèdica Sant Pau-IIB Sant Pau, Hospital de la Santa Creu i Sant Pau, Universitat Autònoma de Barcelona, 08025 Barcelona, Spain; 2 Women and Perinatal Health Research Group, Sant Pau Biomedical Research Institute (IIB-Sant Pau), 08025 Barcelona, Spain; 3 Applied Statistics Department, Universitat Autònoma de Barcelona, 08193 Barcelona, Spain; 4 Primary Care Interventions to Prevent Maternal and Child Chronic Diseases of Perinatal and Developmental Origin Network (RICORS, RD21/0012/0001), Instituto de Salud Carlos III, 28029 Madrid, Spain

**Keywords:** fetal echocardiography, 2D speckle tracking, strain, small for gestational age, fetal growth restriction, aCMQ-QLab

## Abstract

Fetal growth restriction (FGR) is associated with an increased risk of adverse outcomes resulting from adaptive cardiovascular changes in conditions of placental insufficiency, leading to cardiac deformation and dysfunction, which can be evaluated with 2D speckle tracking echocardiography (2D-STE). The aim of the present study was to evaluate whether reduced fetal growth is associated with cardiac left-ventricle (LV) dysfunction, using 2D-STE software widely used in postnatal echocardiography. A prospective longitudinal cohort study was performed, and global (GLO) and segmental LV longitudinal strain was measured offline and compared between FGR and appropriate-for-gestational-age (AGA) fetuses throughout gestation. All cases of FGR fetuses were paired 1:2 to AGA fetuses, and linear mixed model analysis was performed to compare behavior differences between groups throughout pregnancy. Our study shows LV fetal longitudinal strain in FGR and AGA fetuses differed upon diagnosis and behaved differently throughout gestation. FGR fetuses had lower LV strain values, both global and segmental, in comparison to AGA, suggesting subclinical cardiac dysfunction. Our study provides more data regarding fetal cardiac function in cases of placental dysfunction, as well as highlights the potential use of 2D-STE in the follow-up of cardiac function in these fetuses.

## 1. Introduction

Small for gestational age (SGA) fetuses, defined as an estimated fetal weight (EFW) <10th percentile [1], should be distinguished between constitutional small fetuses, with better perinatal outcomes, and fetal growth restricted (FGR) fetuses, associated with signs of fetoplacental dysfunction and with worse perinatal outcomes. In order to perform this distinction, not only does estimated fetal weight (EFW) have to be calculated, but so does fetal growth centile (an EFW below the third percentile or between the third and 10th percentiles with Doppler compromise can be used diagnose FGR), uterine artery Doppler, umbilical artery Doppler, cerebroplacental ratio, and, if possible, maternal angiogenic factors [1].

It is widely reported that abnormal angiogenesis in the placenta can lead to the development of not only FGR, but also maternal preeclampsia (PE) [2], due to impaired remodeling of maternal spiral arteries and placental under perfusion. However, in the last few years, various reports have suggested that placenta-related complications due to angiogenic imbalance may be associated with fetal cardiac remodeling and subclinical dysfunction [3,4,5,6,7,8,9], as well as a higher risk of congenital heart disease (CHD) [10,11,12]. These fetuses are then associated with poorer perinatal outcome, more prenatal death, severe intrapartum fetal distress, and perinatal brain injury [13]. A poorer long-term health outcome has also been described [8,14,15,16,17,18,19], along with impaired neurological and cognitive development, as well as endocrine and cardiovascular disease in adulthood. 

All these facts emphasize the importance of performing good prenatal detection, as well as good pre- and postnatal follow-up of FGR fetuses, taking special interest in the evaluation of the fetal heart. Various ultrasound guidelines [20,21,22] and ultrasound tools are currently available to perform a complete fetal cardiac evaluation. For example, evaluation of cardiac morphometric parameters [23,24,25,26] and conventional Doppler [27,28] are the most globally used, although there are other studies that focused their interest on monitoring fetal cardiac diastolic and systolic function [29,30,31,32,33]. 

In the last few years, with the aim of improving fetal follow-up of FGR fetuses beyond EFW and Doppler assessment, several studies have defined echocardiography strategies to monitor fetal cardiac function and detect cardiac dysfunction in the early stages [6,7,14], with growing interest in 2D speckle tracking echocardiography (2D-STE). A novel ultrasound-based tool first described in adults in 2004 [34] for examining myocardial deformation by measuring strain, 2D-STE identifies speckle patterns of the myocardium to derive strain values, with less angle dependency than other conventional echocardiographic tools [35,36]. This offers advantages for fetal assessment. Fetal strain evaluated with 2D-STE has demonstrated good reproducibility and feasibility [37,38,39,40,41] with a well-defined step-by-step approach [41], and normal reference values for fetal LV strain have been previously published [41], which differ depending on the software or platform used [42,43,44], similar to what happens with other echocardiographic tools [45,46], limiting its current use to research but not clinical practice. 2D-STE has previously been described in FGR fetuses [47]; however, results were heterogeneous and, in some cases, contradictory. 

The aims of our study were to assess the longitudinal behavior of fetal LV longitudinal strain in FGR fetuses, and to compare LV longitudinal strain values between FGR and AGA fetuses, so as to demonstrate cardiac dysfunction in FGR fetuses, using an automated 2D speckle tracking software (aCMQ-QLab) by Philips, which is the most commonly used software postnatally.

## 2. Materials and Methods

### 2.1. Study Population 

Our study population included pregnant women with singleton pregnancies and no evidence of fetal structural cardiovascular disease, who attended the Maternal–Fetal Medicine Department at Hospital de la Santa Creu i Sant Pau in Barcelona, Spain, from June 2018 to December 2021. We prospectively enrolled fetuses with an EFW <10th percentile [1] (FGR group) and appropriate for gestational age (AGA group), defined as estimated fetal weight above the 10th centile. 

Gestational age (GA) was calculated in all pregnancies on the basis of the crown–rump length at first trimester ultrasound [48]. Estimated fetal weight and birth weight centiles were calculated using local reference curves [49]. Differentiation between SGA and FGR was performed following previously reported criteria [1]. All cases of FGR fetuses were paired 1:2 to AGA, according to the gestational age at evaluation.

Patients with maternal age below 18 years, twin pregnancy, structural or chromosomal anomalies, and maternal diseases that could significantly affect the fetal heart, were excluded. During pregnancy, information was collected in order to detect any pregnancy-related conditions associated with remodeling of the fetal heart (i.e., preeclampsia and gestational diabetes). Data such as maternal age at inclusion, race, parity, and BMI were recorded. After delivery, perinatal outcomes such as GA at delivery, mode of delivery, Apgar score, birth weight, birth weight percentile, and neonatal outcomes were recorded. We confirmed that all patients included in the FGR group had a birth weight below the 10th percentile; otherwise, they were excluded from the study. Perinatal mortality was defined as either neonatal death up to the age of 28 days or intrauterine death [50].

The study protocol (IIBSP-CMQ-2017-99) was reviewed and approved by our hospital’s Ethics Committee, and written informed consent was provided by all patients.

### 2.2. Ultrasound Acquisition

Images were acquired using the Affiniti 70G and EPIQ 7W (Philips Healthcare, Andover, MA, USA) ultrasound systems. Fetal routine follow-up was performed in all cases and consisted of fetal biometries, fetal echocardiography, and Doppler parameters for the FGR group. Fetal echocardiography was performed in various examinations, from the day of inclusion to delivery. A 9 MHz sector probe (C9-2, Philips Medical Systems, Andover, MA, USA) was used, and a four-chamber view of the fetal heart was obtained. Specialized obstetricians with experience in fetal cardiology imaging and placental dysfunction performed image acquisition. Stringent criteria for ultrasound acquisition were followed according to previously published recommendations [41]. Care was taken to optimize image quality and acquire images with >80 Hz frames per second (fps). Clip acquisition was performed in the absence of maternal or fetal movements. 

### 2.3. Analysis Protocol

After evaluating clip quality, a clip that included three or four cardiac cycles was analyzed offline by aCMQ-QLab (Philips Medical Systems, Andover, MA, USA). In order to manually determined the cardiac cycle, a previously published protocol was followed [41], selecting the endocardial LV border, tracing the LV endocardium, performing a visual check for tracking quality, and manually correcting if necessary.

**Figure 1:**
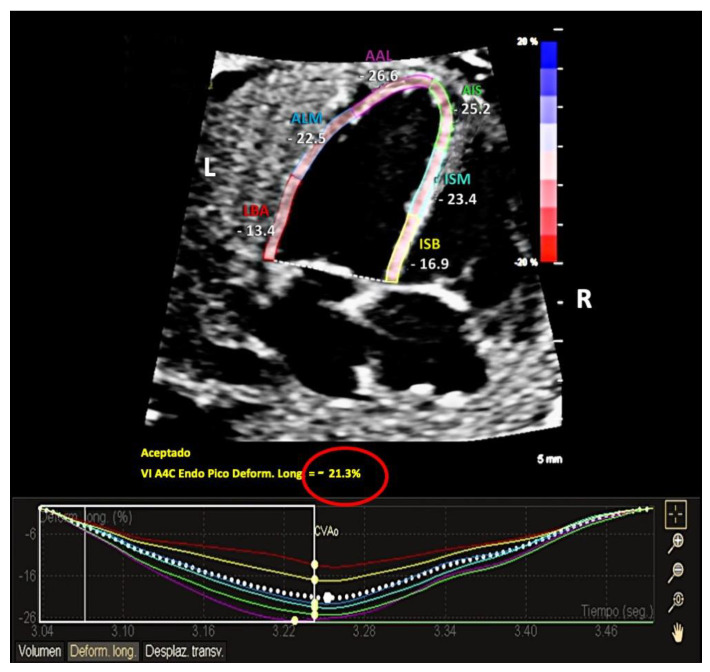
Strain analysis with aCMQ-QLab by Philips. The software automatically delineated the left-ventricular myocardium, providing left-ventricle global longitudinal strain (red circle), as well as individual segment measurements; from left to right: basal segment of left-ventricle wall (LBA), middle segment of left-ventricle wall (ALM), apical segment of left-ventricle wall (AAL), basal interventricular septum (ISB), middle interventricular septum (ISM), and apical interventricular septum (AIS). L, left; R, right. Abbreviations differ from the manuscript, due to software language (Spanish).

Then, global longitudinal strain (GLO) and segmental strain values for the following six segments were automatically provided, as shown in Figure 1: basal interventricular septum (BIS), middle interventricular septum (MIS), apical interventricular septum (AIS), basal segment of left-ventricle wall (BAL), middle segment of left-ventricle wall (MAL), and apical segment of left-ventricle wall (AAL). The software also provides an estimated LV ejection fraction (EF), end-systolic volume (ESV), and end-diastolic volume (EDV).

### 2.4. Statistical Analysis

All clips for FGR fetuses were paired 1:2 to AGA fetuses, according to GA at evaluation. Cases with at least two evaluations during pregnancy were included. All measurements of strain evaluation in FGR fetuses were normalized into Z-values, with a mean of 0 and standard deviation of 1, on the basis of previously published reference curves [41].

For the descriptive analysis, the IBM SPSS Statistics 26 statistical package was used. Variables studied were tested for a normal distribution using the Kolmogorov–Smirnov test. Comparisons between study groups were performed with Student’s *t*-test or χ^2^ test where appropriate, and results are presented as the mean ± standard deviation (SD) or percentage (*n*).

For the longitudinal analysis, a linear mixed model was performed to compare the evolution of Z-value measurements between groups throughout GA, considering subject as the random effect. In all models, the explanatory variables were GA, study group, and the interaction between them. The estimated parameters for each model are presented, and the estimated means for each group throughout GA were plotted.

The significance level was set at 0.05 in all tests. Analysis was performed using SAS v9.4 (SAS Institute Inc., Cary, NC, USA).

## 3. Results

### 3.1. Basic Characteristics of the Study Population

Characteristics of the study population are shown in Table 1. A total of 137 healthy women with AGA fetuses and 45 women with FGR fetuses were included in this study, with a mean gestational age at inclusion time of 30 weeks. Most pregnant women were Caucasian and nulliparous in both groups, with no significant differences in mean maternal age. Five women in the FGR group developed preeclampsia, whereas no cases were identified in the AGA group. As expected, there were differences between AGA and FGR fetuses in terms of GA at delivery (39 vs. 36 weeks, respectively, *p* < 0.001), mode of delivery (81% vaginal delivery vs. 50%, respectively, *p* < 0.050), and birth weight (3315 g vs. 2061 g, respectively, *p* < 0.001). There was one fetal demise at 27 weeks in the FGR group, and no cases in the AGA group. There were four cases (two cases in both groups) for which delivery data were not available due to delivery in another hospital. 

### 3.2. Fetal Ultrasound Assessment

Longitudinal follow-up was performed in all 45 cases of FGR fetuses after inclusion day, with an average of 2.3 echocardiographies per case, obtaining a total of 107 clips: nine clips belonging to SGA fetuses, and 98 clips belonging to FGR fetuses. All cases were matched 1:2 with AGA clips according to GA at ultrasound scan (±1 week) (*n* = 214). 

Strain measurements were feasible in 100% of acquisitions. Mean GA at ultrasound was 32 weeks (25–38) for both groups. As expected, mean EFW at evaluation differed between groups (2003 g in AGA group vs. 1506 g in FGR group, *p* < 0.001). The mean frame rate was 103 fps in both groups for 2D-STE acquisition. 

**Figure 2:**
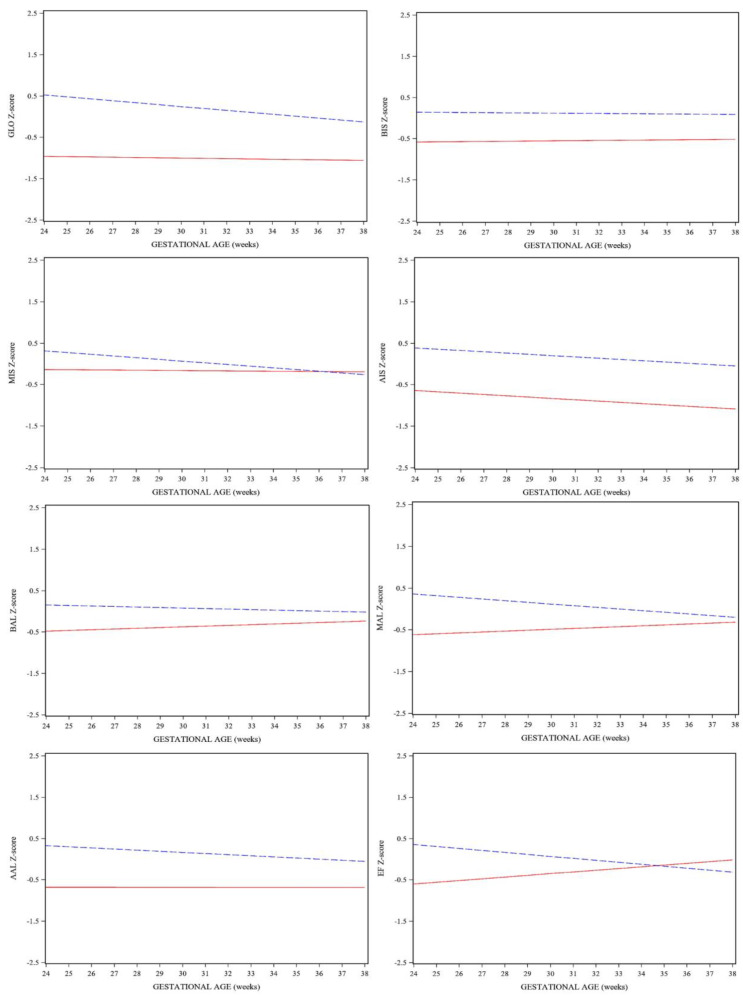
Longitudinal behavior of strain parameters in FGR fetuses (red continuous line) compared to AGA fetuses (blue interrupted line). GLO, global longitudinal strain; BIS, basal interventricular septum; MIS, middle interventricular septum; AIS apical interventricular septum; BAL, basal segment of left-ventricle wall; MAL, middle segment of left-ventricle wall; AAL, apical segment of left-ventricle wall; EF, ejection fraction.

Results of the linear mixed model for repeated measurements of strain evaluation in FGR fetuses are shown in Table 2. Figure 2 shows the longitudinal behavior of LV strain Z-scores in both groups. 

FGR fetuses had a statistically significant lower LV GLO when compared to AGA fetuses (*p* < 0.001) at first evaluation, and this persisted throughout gestation. All segments of LV strain showed similar results, except MIS, where no statistically significant differences were found (*p* = 0.163) and values overlapped at the end of pregnancy.

Our results also showed differences in strain behavior between groups throughout gestation. AGA fetuses had the expected behavior when compared to previously published normal values [41], remaining stable or decreasing slightly as gestation progressed. In the AGA group, GLO, middle, and apical segments showed progressive decline as GA advanced, whereas the basal segments remained stable throughout gestation. FGR fetuses showed a stable behavior in GLO, BIS, MIS, and AAL segments, whereas BAL and MAL showed a progressive increase and AIS showed a progressive decline as gestational age advanced; however, none of these differences were statistically significant.

## 4. Discussion

The objective of this study was to assess longitudinal behavior of fetal LV longitudinal strain in fetuses with an EFW <10th percentile, evaluated by automated 2D speckle tracking software (aCMQ-QLab) and to compare it with AGA fetuses, to demonstrate LV dysfunction in these fetuses throughout gestation. 

Previously published studies proposed the use of several echocardiographic tools to evaluate fetal cardiac function [29,30,31,32,33], showing good results in the evaluation of cardiac dysfunction observed in FGR [5,6,7,9,14,51,52], but with technical limitations, such as fetal apex orientation and angle-dependency. 2D-STE is a new promising tool that solves the angle dependency problem and offers a semiautomated analysis, decreasing intra- and interobserver variability, although some studies have shown discordant results, mostly because of the existence of several commercialized programs with different acquisition protocols and different ultrasound equipment, which make results noncomparable [42,43]. To our knowledge, this is the first study comparing fetal LV longitudinal strain between FGR and AGA fetuses with aCMQ-QLab software, as well as the first study to describe fetal LV strain behavior throughout pregnancy. Good feasibility and reproducibility and normal gestational age-adjusted reference ranges have been previously published by our group using aCMQ-QLab [41]. This software is one of the most commonly used postnatally in our setting, which may allow longitudinal surveillance of strain without intervendor variability, as well as aid in follow-up of fetal cardiac conditions before and after birth. This would eliminate variations when the same parameter is evaluated with different ultrasound equipment or software before and after birth [45,53,54]. 

Our study demonstrated statistically significant lower LV GLO and segment strain values, throughout gestation, in FGR when compared to AGA, indicating that these fetuses have subclinical systolic dysfunction. This finding is similar to other recently published studies [55,56]; however, there are also studies where no differences were found [57,58]. This could probably be explained by the greater knowledge on fetal 2D-STE and technical cardiac imaging improvements in the last few years, since 2D-STE depends on image quality and frame rate in a very sensitive way. The two studies showing no differences in LV longitudinal strain between FGR and AGA were performed earlier (2014 and 2016) than those showing differences (2019 and 2020). 

Our study also showed a different trend in LV strain behavior between AGA and FGR group. While GLO tended to decrease throughout gestation in AGA fetuses, it remained stable in the FGR group. Similar behavior was found in the other LV segments, but these differences were not statistically significant. This behavior in FGR could indicate that the myocardium is less flexible or more rigid due to hypoxia; accordingly, it does not adapt throughout gestation. 

Our cohort mainly comprised mild-FGR fetuses, with only a small number of severe cases. An exploratory analysis was performed using a subclassification of FGR according to local guidelines [1], observing significantly worse values in more severe FGR. Therefore, further studies should be conducted in severe FGR in order to describe strain behavior in this group. Nevertheless, it is noteworthy that, although most of the cases were mild FGR, changes in cardiac function were observed, supporting that, even in mild cases, cardiac function was compromised.

Cardiac LV dysfunction, observed in FGR fetuses as abnormal LV longitudinal strain values, could be explained by cardiac remodeling observed in placental insufficiency, which leads to an increase in placental vascular resistance and chronic fetal hypoxia. This can increase LV and RV afterload, as well as cause remodeling, leading to a more ‘globular’ and rigid heart [9,51]. The fetal response to hypoxia is shown as a decrease in cerebral vascular resistance with a consequent reduction in LV afterload and redistribution of blood flow toward the LV through the foramen ovale, increasing LV preload and favoring perfusion of the fetal heart, brain, and adrenal glands [59]. If hypoxia continues, compensatory mechanisms might become insufficient, causing cardiac deformation and systolic and diastolic abnormalities.

Previously published studies have shown discordant results when evaluating fetal strain in FGR fetuses, mostly due to heterogeneous study populations, different GA between groups at ultrasound evaluation, or absence of postnatal confirmation of the diagnosis [47]. Our study had various strengths in this regard. We followed strict previously published criteria for 2D-STE evaluation obtained by aCMQ-QLab [41]. Our study population and GA at ultrasound acquisition were homogeneous and comparable between groups. We recorded high-frame-rate acquisitions with very high temporal resolution in both groups. We also confirmed FGR with birth weight and birth weight percentile in all patients, since calculation of EFW by ultrasound usually overestimates the actual fetal weight, especially in SGA fetuses [60,61]. 

Some limitations of this study should also be considered. Firstly, since commercial software provides different results due to the use of different algorithms [42], it is unlikely that these results can be transferred to other software vendors. Another relevant limitation is that, at this moment, this novel software only contemplates LV analysis, although studies are being conducted to validate evaluation of the RV, limiting its clinical application to some fetal conditions. Secondly, we only assessed longitudinal strain in a four-chamber view, which is the only evaluation currently validated by aCMQ-QLab. It is important to bear in mind that circumferential and axial myocardial fibers should also be ideally evaluated, although longitudinal myocardial fibers are affected first in most cardiac conditions. Lastly, despite the large number of examinations performed in our study, the subgroup sample size might still be limited in making robust conclusions regarding our outcomes, since most of our FGR fetuses had mild hypoxia; thus, further studies are needed to describe strain behavior in cases of severe hypoxia. 

2D-STE by aCMQ-QLab is a new technique, currently used in research, and this was the first study to evaluate cardiac strain in FGR fetuses using this novel software. 

On the basis of our research, we found that the evaluation of longitudinal LV strain in FGR fetuses can be a useful tool for monitoring these fetuses and assessing fetal compromise. However, it should not be solely relied upon as a prenatal predictor of FGR due to the various conditions during pregnancy that can affect cardiac function; additionally, the estimation of fetal weight is the most reliable, reproducible, and readily available method for detecting fetuses at risk of fetal growth restriction. 

Our research showed evidence of subclinical systolic dysfunction in FGR fetuses from the initial evaluation, which persisted throughout gestation. As our study primarily focused on mild FGR fetuses, with only a few severe cases, it is unlikely that these parameters can predict acute fetal and neonatal outcomes. 

Nevertheless, further studies should be conducted to gain a better understanding of strain behavior, determine whether longitudinal LV strain evaluation can serve as a reliable tool to predict perinatal or fetal outcomes in the mid and long term, and evaluate its use in clinical practice. This would allow for longitudinal pre- and postnatal surveillance, avoiding intervendor variability.

## 5. Conclusions

In conclusion, LV fetal longitudinal strain in FGR and AGA fetuses is different upon diagnosis and behaves differently throughout gestation. FGR fetuses have lower LV strain values, both global and segmental, in comparison to AGA, suggesting subclinical cardiac dysfunction. LV strain can be a valuable complementary tool in monitoring the cardiac function of these fetuses throughout gestation and postnatally, avoiding intervendor variability by using the same provider. 

## Figures and Tables

**Table 1:** Basic characteristics of the study population.

Variable	FGR (*n* = 45)	AGA (*n* = 137)	*p*-Value
Clinical characteristics			
Maternal age, years	34 ± 5	33 ± 5	0.551
Caucasian	80 (36)	72 (100)	>0.05
Body mass index at inclusion, kg/m^2^	24 ± 5.07	22 + 3.04	0.110
Nulliparity	60 (27)	75 (103)	>0.05
Pregnancy outcome			
Preeclampsia	11 (5)	0 (0)	<0.05
GA at delivery	36 ± 3	39 ± 1.03	0.001
Vaginal delivery	50 (23)	81 (111)	<0.05
Caesarean delivery	44 (20)	17 (24)	<0.05
Birth weight, g	2061 ± 619	3315 ± 338	0.001
Birth weight centile	2 ± 2	42 ± 25	0.001

Data are shown as the mean ± standard deviation or percentage (*n*).

**Table 2:** Summary of linear mixed models for repeated measures of longitudinal strain values in FGR.

Variable	Group	Fixed			Random	
		Intercept (Z)	GA (Weeks) (Z)	GA (Weeks) (Z)	Intercept (SE)	Residual (SE)
GLO	−1.2147 (0.1228)	0.208 (0.049)	−0.0466 (0.0098)	0.0396 (0.0317)	0.0849	0.6668
BIS	−0.6659 (0.1325)	0.117 (0.0528)	−0.00385 (0.0112)	0.00852 (0.0356)	0.0681	0.8730
MIS	−0.1978 (0.1415)	0.03415 (0.0567)	−0.0409 (0.0112)	0.0368 (0.0360)	0.1231	0.8548
AIS	−1.033 (0.1399)	0.1753 (0.0568)	−0.0310 (0.0096)	−0.00068 (0.0317)	0.1947	0.6155
BAL	−0.4303 (0.1270)	0.0703 (0.0503)	−0.0125 (0.0117)	0.0298 (0.03644)	0.01195	0.9664
MAL	−0.5563 (0.1288)	0.0865 (0.0511)	−0.0399 (0.0113)	0.0613 (0.0357)	0.0417	0.8977
AAL	−0.8246 (0.0347)	0.1415 (0.0504)	−0.0272 (0.0110)	0.0270 (0.0347)	0.0491	0.8428
EF	−0.3457 (0.1382)	0.0304 (0.0553)	−0.0477 (0.0111)	0.0895 (0.0357)	0.1060	0.8495

The table shows the parameter estimation and standard error (SE). GA, gestational age; GLO, global longitudinal strain; BIS, basal interventricular septum; MIS, middle interventricular septum; AIS apical interventricular septum; BAL, basal segment of left-ventricle wall; MAL, middle segment of left-ventricle wall; AAL, apical segment of left-ventricle wall; EF, ejection fraction; Z, Z-score.

## Data Availability

The data presented in this study are available on request from the corresponding author. The data are not publicly available due to privacy restrictions.
